# Force of infection of Middle East respiratory syndrome in dromedary camels in Kenya

**DOI:** 10.1017/S0950268819001663

**Published:** 2019-09-24

**Authors:** E. G. Gardner, S. Kiambi, R. Sitawa, D. Kelton, J. Kimutai, Z. Poljak, Z. Tadesse, S. Von Dobschuetz, L. Wiersma, A. L. Greer

**Affiliations:** 1 University of Guelph, Guelph, Ontario, Canada; 2 Food and Agriculture Organization of the United Nations, Nairobi, Kenya; 3 Directorate of Veterinary Services, Nairobi, Kenya; 4 Food and Agriculture Organization of the United Nations, Nairobi, Italy

**Keywords:** Dromedary camels, emerging infections, force of infection, MERS-CoV, reservoir

## Abstract

Middle East respiratory syndrome coronavirus (MERS-CoV) is a zoonotic disease transmitted from dromedary camels to people, which can result in outbreaks with human-to-human transmission. Because it is a subclinical infection in camels, epidemiological measures other than prevalence are challenging to assess. This study estimated the force of infection (FOI) of MERS-CoV in camel populations from age-stratified serological data. A cross-sectional study of MERS-CoV was conducted in Kenya from July 2016 to July 2017. Seroprevalence was stratified into four age groups: <1, 1–2, 2–3 and >3 years old. Age-independent and age-dependent linear and quadratic generalised linear models were used to estimate FOI in pastoral and ranching camel herds. Models were compared based on computed AIC values. Among pastoral herds, the age-dependent quadratic FOI was the best fit model, while the age-independent FOI was the best fit for the ranching herd data. FOI provides an indirect estimate of infection risk, which is especially valuable where direct estimates of incidence and other measures of infection are challenging to obtain. The FOIs estimated in this study provide important insight about MERS-CoV dynamics in the reservoir species, and contribute to our understanding of the zoonotic risks of this important public health threat.

## Introduction

Middle East respiratory syndrome coronavirus (MERS-CoV) is an emerging zoonotic infection that spills over from the dromedary camel reservoir to humans. It was first reported in 2012, and causes severe respiratory disease in humans with a case fatality rate of approximately 35% [1]. The virus is endemic in dromedary camels across the Middle East, Africa and parts of South Asia [2]. However, zoonotic transmission from camels to humans has been reported only in the Middle East [3]. Differences in human–animal contact patterns, camel husbandry and production, and viral genetics have all been proposed as possible reasons for the apparent disparity in regional zoonotic risk [3]. Little is known about MERS-CoV dynamics within the dromedary camel reservoir, and what impact these dynamics might have on the risk of zoonotic transmission.

Although seroprevalence is high in many dromedary populations, point prevalence of infection appears to be variable [4–8], which makes assessing zoonotic risk challenging. MERS-CoV incidence, an important measure of infection dynamics and transmission risk, is difficult to estimate in dromedary camels because the infection is often subclinical or very mild and thus easy to miss [9]. Previous studies have suggested that suckling and recently weaned calves have the highest incidence, shed more virus and are the drivers of MERS-CoV circulation among dromedaries. As a result, these animals are presumably a higher risk source for zoonotic transmission, whether through direct contact or indirectly through localised transfer to the dam's milk and subsequent consumption of contaminated raw camel milk [10, 11]. These conclusions are drawn primarily from a small number of longitudinal studies [6–12] and age-stratified prevalence data, where the trend points towards low seroprevalence and higher prevalence of active infection in calves as compared to older age groups [6–13].

The force of infection (FOI) is defined as ‘the rate at which susceptible individuals become infected per unit time’, or the probability that a susceptible individual will become infected per unit time, and depends on the number of infectious individuals in a population and their contact rate with susceptible individuals [14]. FOI can be estimated from disease prevalence data [15], and the product of FOI and the number of susceptible individuals will yield estimated incidence [16]. Previous studies on other pathogens have used prevalence data to estimate FOI and derive incidence in a population of interest [17], and FOI has been used as a metric for comparison of disease burden spatially and temporally [18, 19]. To our knowledge, this is the first study to investigate FOI for MERS-CoV in dromedary camels. FOI can also be used to estimate the reproductive number of the pathogen in a population (the average number of secondary infections resulting from the introduction of an infectious individual into a completely susceptible population) [20, 21], and as a parameter in disease transmission models [22].

Improving our understanding of MERS-CoV dynamics in camels raised outside of the Middle East will contribute towards a greater understanding of the virus in the reservoir host, and may provide points of comparison for viral dynamics in camels in the Middle East where zoonotic transmission is ongoing. Age-stratified seroprevalence data were collected from dromedary camels in five provinces in Kenya. The objective of this study was to estimate the FOI among dromedary camels raised in pastoral and ranching production systems in Kenya.

## Methods

### Cross-sectional survey

**Fig. 1.**
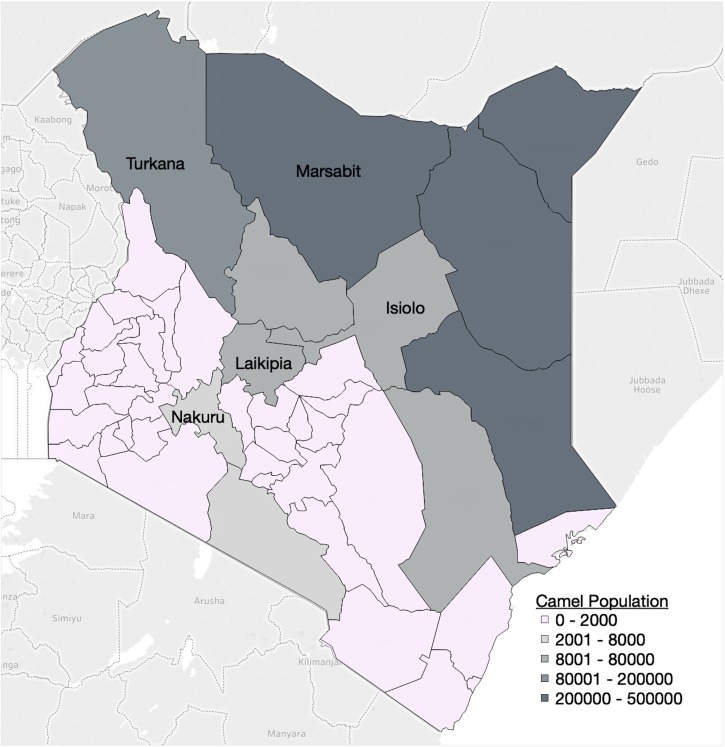
Map of Kenya with counties shaded according to camel population. The five counties included in the cross-sectional MERS-CoV camel survey are labelled. Map adapted from Gikonyo *et. al*. (2018) [23] using Tableau^©^ Desktop 10.5.

Age-stratified seroprevalence data were collected as part of a cross-sectional survey of MERS-CoV in dromedary camels in Kenya, conducted by the Directorate of Veterinary Services of Kenya and the Food and Agricultural Organization of the United Nations between July 2016 and July 2017. The study area consisted of the five main camel-producing counties in Kenya, as determined previously [23], excluding those where travelling and sampling were not possible due to security concerns (Fig. 1). A multi-level sampling strategy was employed. The overall sample size was 1421 camels from 83 herds, with a probability sampling of herds from each county (Table 1). Approximately one-third of sublocations (the lowest administrative unit) were randomly selected from each county. A list of households and the number of camels owned in each selected sublocation was used as the sampling frame. The number of herds sampled in each sublocation was proportional to the total number of herds in that administrative unit. Age was divided into four categories: 0–1, 1–2, 2–3 and >3 years of age. Within each herd, camels were sampled conveniently with attention that all age groups and both sexes were represented. All camels were sampled in herds 10 or smaller, 10–20 camels were sampled in herds between 11 and 50 animals, and 20 camels were sampled in herds larger than 50.

**Table 1.** Estimated camel population and proportion sampled by county

County	*N* sampled	Camel population	% Sampled
Isiolo	403	68 000	0.59
Laikipia	181	8000	2.26
Marsabit	370	200 000	0.19
Nakuru	50	1000	5.0
Turkana	417	800 000	0.05

Estimated camel population was sourced from [23] and (Stephen Gikonyo, personal communications).

Individual animal data collected included age and sex. Herd-level variables included production system: pastoral herds are defined primarily as camels that are raised extensively; ranch herds are raised semi-intensively or intensively. Differences between pastoral and ranch production systems also exist in how breeding stock are sourced, migration and marketing of camels and camel products. Other herd-level factors collected included herd size; survey respondent's role; presence/absence of a perimeter wall; interaction with other camels, wildlife and other domestic species; water source; and whether the camels were confined at night.

Blood was collected by venipuncture, serum was separated by a portable centrifuge on site after clotting and stored at 20 °C for shipment and storage until testing at the Central Veterinary Laboratory in Kenya. Anti-MERS-CoV (IgG) antibodies were tested using the EUROIMMUN ELISA Camel test kit [24].

### Data analysis and FOI estimation

R version 3.5.1 [25] was used for all data cleaning, descriptive analysis of MERS-CoV seroprevalence and generation of FOI estimates. Three methods for estimating FOI were compared. A catalytic model first described by Muench [26] that estimates a constant FOI was compared to two age-dependent models, one in which FOI is a linear function, and a model in which FOI is a quadratic function of age groups. Generalised linear models were used as a statistical framework for each of the three models, adapted from Hens *et al*. [15]. Data were organised into age groups by herd. The midpoint of each age category was used. For the category >3 years, 3.5 years was used due to a lack of demographic information on the upper bound of this category. Residual *vs.* leverage plots were used to identify highly influential observations. AIC was used to compare the models for best fit.

## Results

### Descriptive results of cross-sectional survey

The cross-sectional seroprevalence study included samples from 1421 dromedary camels from 83 herds in five counties. The overall individual-level seroprevalence was 63.7% (95% confidence interval 61.2–66.2%). At the herd level, 86.7% of herds had at least one seropositive camel (95% CI 79.5–94.0%) while the mean seroprevalence within all herds was 57.6% (95% CI 50.8–64.4%). Table 2 shows seroprevalence by county, age, sex and production system. Differences in seroprevalence can be seen across the different factors, perhaps most strikingly between counties and production systems. Two counties (Nakuru and Laikipia) contained all ranching herds, while only a few pastoral herds were sampled in Laikipia, and none in Nakuru. The seroprevalence levels by county and production system are consistent with this clustering. Furthermore, ranching herds tended to be smaller. The average herd size was 77 camels, with no herds having more than 200 camels. Pastoral herds by comparison had an average herd size of 128 camels. Ranch had less contact with other animals including camels, livestock and wildlife (data not shown). Conversely, age and sex were similarly distributed among the two production systems. For these reasons and due to a large difference in seroprevalence, production system appeared to be an important characteristic in camel raising and MERS-CoV transmission, and was the basis for exploring FOI separately in ranching and pastoral herds.

**Table 2.** Camel seroprevalence by county, age and sex

	No. Positive	No. sampled	% Positive (95% CI)
County			
Isiolo	313	403	77.7 (73.6–81.7)
Laikipia	27	181	14.9 (9.7–20.1)
Marsabit	274	370	74.1 (69.6–78.5)
Nakuru	7	50	14.0 (4.4–23.6)
Turkana	284	417	68.1 (63.6–72.6)
Age			
<1	60	157	38.2 (30.6–45.8)
1–2	107	223	48.0 (41.4–54.5)
2–3	95	156	60.9 (53.2–68.6)
>3	643	885	72.7 (69.7–75.6)
Sex			
Female	752	1129	66.6 (63.9–69.4)
Male	153	292	52.4 (46.7–58.1)
Production system			
Pastoral	892	1292	69.0 (66.5–71.6)
Ranching	13	129	10.1 (4.9–15.3)

### Force of infection

**Fig. 2.**
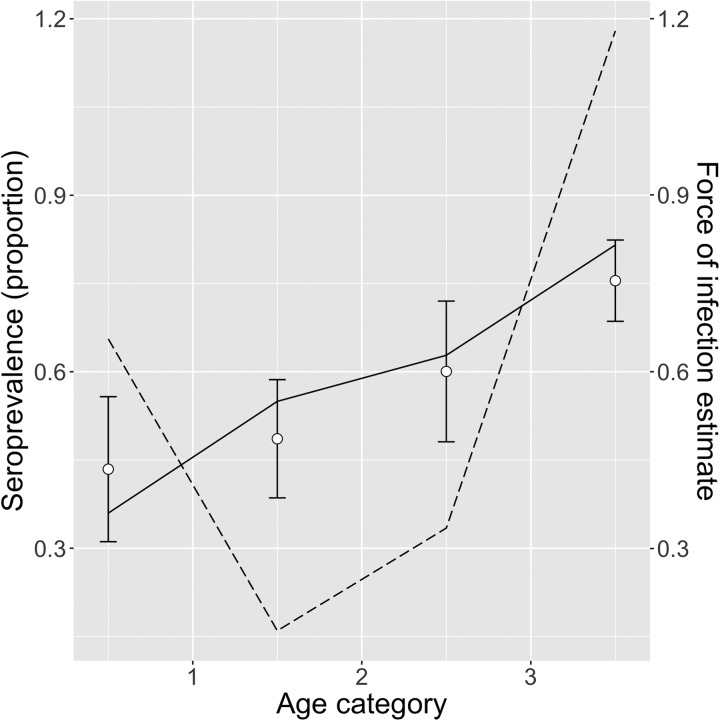
Quadratic model of pastoral herds: mean observed herd seroprevalence with 95% CI (white circles with error bars), predicted seroprevalence (solid black line) and estimated force of infection (dotted black line) for pastoral camel herds in Kenya.

**Fig. 3.**
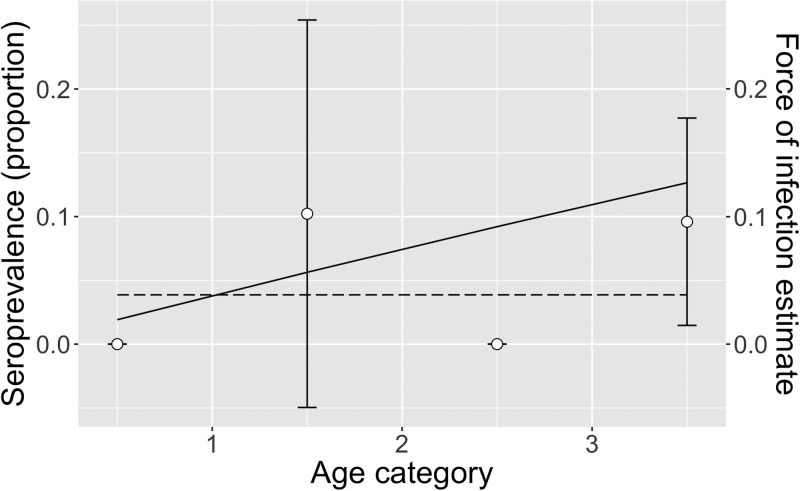
Exponential model of ranch herds: mean observed herd seroprevalence with 95% CI (white circles with error bars), predicted seroprevalence (solid black line) and estimated force of infection (dotted black line) for ranching camel herds in Kenya. No camels tested positive among the <1 and 2–3 year age groups, therefore no error bars were calculated.

Table 3 shows the FOI estimates for pastoral and ranching herds for constant, linear and quadratic models, along with each model's AIC. FOI was consistently lower for ranching herds than pastoral herds. According to AIC values, the quadratic model was the best fit for pastoral herds, while the simple catalytic model was the best fit for ranching herds. Among pastoral herds, FOI was higher in the youngest and oldest age groups (0.69 and 1.05, respectively), while the middle age groups had lower FOIs. Among camel herds raised in a ranch setting, the age-independent FOI was estimated to be 0.039. Figures 2 and 3 show the observed mean seroprevalence for each age group with 95% confidence intervals as well as the model outputs of expected seroprevalence and best fit FOI for pastoral and ranching herds. Table 4 lists the sample size (number of observations) and seroprevalence per production system and age group.

**Table 3.** Force of infection estimates and AIC values of three models for pastoral and ranching camel herds in Kenya

	Pastoral herds		Ranching herds	
Model	FOI estimate	AIC	FOI estimate	AIC
Constant FOI	0.47	790.58	0.039	34.24
Linear FOI				
<1 year	0.626	777.38	0.060	35.83
1–2 years	0.471		0.040	
2–3 years	0.316		0.021	
3+ years	0.161		0.001	
Quadratic FOI				
<1 year	0.686	764.74	0.117	36.75
1–2 years	0.136		−0.104	
2–3 years	0.259		−0.032	
3+ years	1.054		0.334

**Table 4.** Number of observations and seroprevalence per age group in pastoral and ranching herds

	Pastoral		Ranching	
Age	No. sampled	% Positive (95% CI)	No. sampled	% Positive (95% CI)
<1 year	143	42.0 (33.9–50.0)	14	0
1–2 years	203	51.2 (44.4–58.1)	20	15 (−0.6 to 30.6)
2–3 years	143	66.4 (58.7–74.2)	13	0
>3 years	803	78.8 (76.0–81.7)	82	12.2 (5.1–19.3)

Examination of residual *vs.* leverage plots for pastoral herds identified three observations that were possible outliers, with high standardised Pearson residual, high leverage or both. FOI model outputs did not appreciably change after stepwise removal of the three observations (data not shown); therefore, reported results include the complete dataset.

## Discussion

Among pastoral herds, the highest FOI was among the oldest age group (>3 years old). This is in contrast to previous surveys which have found the highest proportion of shedding to be among calves [6], suggesting that the youngest age group are at highest risk of infection [12]. There are a few possible reasons why this FOI analysis identified the oldest age group as having the highest risk of infection based on model AIC. Questions remain as to whether antibodies to MERS-CoV wane after first infection, and if and to what degree camels are susceptible to reinfection [3–12, 13–27]. The FOI analysis presented in this paper used serological data, not virological results. If seroconversion does not necessarily occur after the first infection, or antibodies wane following a single infection, then the risk profile seen in this analysis as well as in previous studies may reflect a more complex immunological picture, where, for example, seroconversion occurs after subsequent infections (occurring in older camels) [13–28]. Furthermore, the oldest age group has a much larger age range than the others, and therefore represents a greater cumulative risk. Although more detailed age demographic data were not collected during this survey, previous work in pastoral herds in northern Kenya has reported that breeding females live to an average of 19 years, and that breeding bulls (1–4 in each herd) are culled at about 17 years old [29]. Male calves, however, are either slaughtered shortly after birth, or at about 4 years of age [30]. Previous research has shown that dams of suckling calves also shed MERS-CoV [11]. Female camels typically have their first calf between 4 and 5 years of age [31], placing dams, potentially one of the higher-risk demographic groups, in the oldest age group. The lack of detailed age demographics of the herds sampled in the survey precluded an estimation of the midpoint of the oldest age category, but it is presumably higher than the 3.5 years used in this study. A higher age midpoint would result in different model FOI estimations and AIC values.

The production system in which camels were raised appeared to have an important impact on the risk of MERS-CoV infection. The FOI was consistently lower in ranching herds than pastoral herds, by a factor of at least 10 when comparing the models with the best fit. This is consistent with previous studies, where the production system has been demonstrated to be a significant factor in MERS-CoV seroprevalence in camels [28]. However, a previous study of camel herds in Laikipia County, Kenya found no statistical difference in seroprevalence based on herd management strategy [32]. There are numerous factors that clustered by production system which may contribute to the difference in infection risk, including county. Camel density at the county level is lower where ranching herds are found [23], and given that MERS-CoV is transmitted by close contact between camels, regional density, which could be related to production system, may be a factor that impacts virus transmission. Indeed, regional camel density was found to correlate with seroprevalence in a previous study of Kenyan camel herds, although production systems clustered geographically in this study as well [33]. Other factors that clustered with production system were related to herd management and husbandry. According to data collected in this survey, pastoral herds only used communal water sources, while ranching herds mostly kept herds within a fenced area (data not shown). Both of these management characteristics affect contact with other (potentially infectious) animals. Indeed, all herds in this study that lacked a perimeter fence had daily contact with other camels, while fenced herds did not. Production system may impact infection risk for many reasons, including herd-level factors and county-level factors. The data from pastoral herds best fit an age-dependent model, while ranching herd data fit the age-independent model. This may reflect differences in herd management and structure, or this difference may be due to a small sample size of ranching herds.

The FOI of MERS-CoV estimated in this study can be interpreted as a measure of the burden of infection by age group. With a known camel population size, estimated incidence can be derived, which is especially useful when incidence cannot be measured directly, such as for a subclinical infection like MERS-CoV which is challenging and unrealistic to monitor in camel populations, yet is important from a zoonotic risk perspective. FOI can be used as a parameter in MERS-CoV transmission models to explore infection dynamics that are otherwise difficult to measure. For example, models using FOI estimates can investigate questions around camel immunity to MERS-CoV, validated with field data.

The shape of the FOI curves is quite different among the constant, linear and quadratic models. In order to determine whether any observations may be exerting a strong influence on the model, the standard Pearson residual was plotted against leverage, and possible outliers were identified and removed piecewise from the models. The model outputs did not qualitatively change as a result of removing the observations with higher residual and leverage values, indicating that the shape of the FOI estimates was dictated by the model rather than any outliers.

This study had a number of limitations. The cross-sectional survey on which this study is based used multi-stage probability sampling. However, logistical constraints and convenience sampling led to a wide variation in the sampling fraction by county (Table 1), which may introduce bias in the seroprevalence estimates. Inaccurate seroprevalence estimates in turn may affect the validity of FOI calculations. Furthermore, the results of the cross-sectional survey revealed that pastoral and ranching herds had considerable differences in seroprevalence, as well as a number of distinctions in herd management practices which may impact transmission risk. However, the sample size for ranching herds (10 herds) was quite a bit smaller than for pastoral herds (73 herds). A larger sample size may provide more insight regarding these differences, and provide a more robust FOI estimation.

One of the assumptions of the FOI estimation methods used in this study is that antibodies are lifelong. Field surveys have consistently shown that seroprevalence increases with age [34], an indication that antibodies are long-lasting. On the other hand, a number of observational studies have demonstrated that antibodies may not confer complete protection, and that dromedaries may be susceptible to reinfection despite having previously seroconverted [12–35, 36]. Given that this study assumed seroconversion to be a proxy for infection, the interpretation of FOI should be for first infections only. The risk for reinfection remains unknown, and the total number of infections (first and subsequent) may be underestimated.

While pastoral camels <1 year old had a relatively high FOI, as expected, the seroprevalence used to calculate this estimate may include young camels with maternal antibodies. Passive immunity may therefore be confounded with natural infection, resulting in an overestimate of the FOI.

**Fig. 4.**
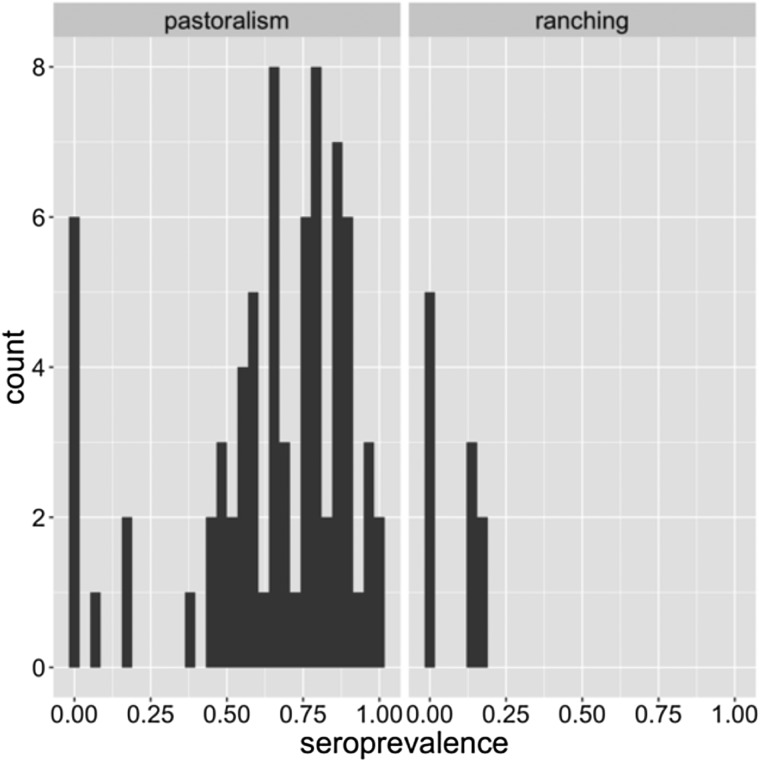
Distribution of within-herd seroprevalence in pastoral and ranching herds.

Figure 4 is an illustration of the distribution of within-herd seroprevalence among pastoral and ranching herds. Especially among pastoral herds, the distribution is wide (0–100%) and bimodal, with a cluster of low seroprevalence herds, and a large cluster of high (>50%) seroprevalence herds. This distribution indicates that perhaps there are other factors within pastoral herds that also influence transmission risk. For example, herd density, calving management, geographical location and herd movement patterns may all influence the risk of transmission and exposure to MERS-CoV.

This study examined FOI separately in pastoral and ranching herds. Qualitatively different risks of infections were estimated for the two types of production systems. These FOI estimates provide another perspective on MERS-CoV dynamics and a metric for infection risk in the dromedary camel reservoir, which can be used to estimate incidence, as a parameter in transmission models, and as a point of comparison between different camel populations. Within a one-health context, the results described here can also help to inform zoonotic transmission risk assessment, human surveillance and other MERS-CoV activities at the camel–human interface.
